# Supplementary material for: Ligand-switchable nanoparticles resembling viral surface for sequential drug delivery and improved oral insulin therapy
Source: Nat Commun. 2022 Nov 4;13:6649. doi: 10.1038/s41467-022-34357-8 (PMC9636268; doi:10.1038/s41467-022-34357-8)
Supplement: Supplementary file 1 — Supporting Information [file 41467_2022_34357_MOESM1_ESM.pdf]

# Supplementary Information

## Ligand-switchable nanoparticles resembling viral surface for sequential drug delivery and improved oral insulin therapy

*Tiantian Yang*[1,2], *Aohua Wang*[1,2], *Di Nie*[1,2], *Weiwei Fan*[1,2], *Xiaohe Jiang*[1,2], *Miaorong Yu*[1,2], *Shiyan Guo*[1], *Chunliu Zhu*[1], *Gang Wei*[3*], *Yong Gan*[1,2,4*]

[1]State Key Laboratory of Drug Research, Shanghai Institute of Materia Medica, Chinese Academy of Sciences, Shanghai 201203, China

[2]University of Chinese Academy of Sciences, Beijing 100049, China

[3]Key Laboratory of Smart Drug Delivery, Ministry of Education, Department of Pharmaceutics, School of Pharmacy, Fudan University, Shanghai 201203, China

[4]NMPA Key Laboratory for Quality Research and Evaluation of Pharmaceutical Excipients, National Institutes for Food and Drug Control, Beijing 100050, China

Correspondence and requests for materials should be addressed to G.W. (email: weigang@shmu.edu.cn) or to Y.G. (email: ygan@simm.ac.cn)

## Table of Contents

**Supplementary Methods**

Supplementary Method 1. NMR characterization of Pep

Supplementary Method 2. The spectral properties of Edans and Dabcyl

Supplementary Method 3. Hemolytic effect test

Supplementary Method 4. Synthesis of $NH_2$-PEG-Gal polymers

Supplementary Method 5. *In vitro* stability and insulin release studies

Supplementary Method 6. Mucus penetrating studies

Supplementary Method 7. Cytotoxicity studies

Supplementary Method 8. Cellular uptake studies of nanoparticles at different pH

Supplementary Method 9. Cellular uptake studies and transcytosis of free insulin

Supplementary Method 10. Cellular uptake mechanism of Pep/Gal-PNPs

Supplementary Method 11. ASGPR expression studies

Supplementary Method 12. Pulse-chase studies of insulin-loaded Pep/Gal-PNPs on hepatocytes

Supplementary Method 13. Hepatic insulin availability studies of Pep/Gal-PNPs

**Supplementary Figures**

Supplementary Fig. 1. $^1$H NMR spectrum of Pep

Supplementary Fig. 2. FRET pair analysis

Supplementary Fig. 3. The hemolytic effect of Pep at different pH

Supplementary Fig. 4. $^1$H NMR spectra of Gal, PEG, and PEG-Gal polymers

Supplementary Fig. 5. Detection of Gal-PNPs via AFM

Supplementary Fig. 6. The hemolytic effects of PNPs and Pep/Gal-PNPs at different pH

Supplementary Fig. 7. *In vitro* stability of Pep/Gal-PNPs

Supplementary Fig. 8. *In vitro* insulin release from Pep/Gal-PNPs

Supplementary Fig. 9. 3D images showing the distribution of Pep/Gal-PNPs in the mucus layer

Supplementary Fig. 10. Cytotoxicity of Pep/Gal-PNPs on Caco-2 cells under different pH conditions

Supplementary Fig. 11. The hemolytic effects of CPP and CPP/Gal-PNPs at different pH

Supplementary Fig. 12. The cellular uptake of nanoparticles by Caco-2 cells at

different pH
Supplementary Fig. 13. The cellular uptake and transcytosis of free insulin
Supplementary Fig. 14. Cellular uptake mechanism of Pep/Gal-PNPs at different pH
Supplementary Fig. 15. Transepithelial electrical resistance (TEER) of Caco-2 cells after treatment with different formulations
Supplementary Fig. 16. The expression of ASGPRs on liver cells
Supplementary Fig. 17. Cytotoxicity of Pep/Gal-PNPs on LO2 cells under different pH conditions
Supplementary Fig. 18. CLSM images of a) PNPs and b) CPP/Gal-PNPs binding to LO2 cells
Supplementary Fig. 19. Pulse-chase analysis of p-AKT expression in LO2 cells
Supplementary Fig. 20. Intestinal absorption of PNPs at different pH
Supplementary Fig. 21. Quantitative analysis of accumulation ratio of PNPs, CPP/Gal-PNPs, and Pep/Gal-PNPs in major organs.
Supplementary Fig. 22. *In vivo* distribution of PNPs
Supplementary Fig. 23. CLSM images of colocalization of PNPs with ASGPRs in liver sections
Supplementary Fig. 24. Blood glucose level versus time profiles in type I diabetic rats
Supplementary Fig. 25. Portal serum insulin level versus time profiles in type I diabetic rats treated with different formulations
Supplementary Fig. 26. Simultaneous measurement of portal (Po) and peripheral (Pe) a) serum insulin level versus time profiles and b) the total area under the serum insulin level versus time curve in type I diabetic rats.
Supplementary Fig. 27. The H&E staining images of liver and intestine sections from healthy rats treated with PBS

**Supplementary Tables**

Supplementary Table 1. Characterization of nanoparticles
Supplementary Table 2. Pharmacodynamic parameters of different insulin formulations following oral or subcutaneous administration to diabetic rats
Supplementary Table 3. Measurement of portal serum insulin levels in diabetic rats following oral or subcutaneous administration of different insulin formulations

**Supplementary Method 1. NMR characterization of Pep.**

The Pep was dissolved in the DMSO-d6 at a concentration of 1 mg $mL^{-1}$. Then, the Pep we characterized by $^{1}H$ NMR spectroscopy.

**Supplementary Method 2. The spectral properties of Edans and Dabcyl.**

The Edans and Dabcyl were dissolved in DMSO and diluted with PBS at pH 3.0, 5.0, 6.0, 6.8, 7.0, 7.4 and 8.0 to 0.1 μg $mL^{-1}$. Then the emission spectra of Edans were detected from 370 nm to 700 nm with an excitation wavelength at 340 nm and the absorption spectra of Dabcyl were detected from 400 nm to 700 nm by a microplate reader.

**Supplementary Method 3. Hemolytic effect test.**

The rat blood was collected and then centrifuged at 1503 × g for 10 min. The red blood cells (RBCs) were obtained and washed three times with fresh PBS. Then the RBCs were diluted with PBS at pH 6.0, 6.5, 7.0, 7.5, 8.0 respectively to 2% (w/v). The Pep and CPP were also dissolved in PBS of different pH to a final concentration of 0.5 mg $mL^{-1}$. Then, 100 μL Pep or CPP solution was added to an equal volume of RBCs suspension and incubated at 37 °C for 1 h. After centrifugation, the supernatants were collected and the absorbance at 570 nm was measured using a microplate reader. The RBCs that were treated with PBS and 1% Triton X-100 were set as control groups, and the hemolysis level of Pep and Gal was measured using the following formula:

$$Hemolysis\% = \frac{A(sample) - A(PBS)}{A(Triton) - A(PBS)} \times 100 \quad (1)$$

Furthermore, the hemolytic effect of nanoparticles was studied using the method mentioned above and the concentration was set as 1 mg $mL^{-1}$.

**Supplementary Method 4. Synthesis of $NH_2$-PEG-Gal polymers.**

The $NH_2$-PEG-Gal polymers were synthesized via the condensation reaction of the carboxyl group of galacturonic acid with the amino group of $NH_2$-PEG-$NH_2$ polymers. In brief, galacturonic acid (20 mg, 0.1 mmol), N-hydroxysuccinimide (NHS, 58 mg, 0.5 mmol) and 1-ethyl-3-(3-dimethylaminopropyl)-carbodiimide (EDC, 95 mg, 0.5 mmol) were dissolved in dimethylsulfoxide (DMSO, 0.5 mL). After stirring for 15 min at room temperature, $NH_2$-PEG-$NH_2$ (100 mg, 0.1 mmol) polymers were added to

the mixture and continued to stir overnight. The unreacted EDC, NHS, and galacturonic acid were removed by dialysis (Mw cutoff: 3500 Da) against deionized water and the final solution was lyophilized to obtain the $NH_2$-PEG-Gal polymers. The polymers were dissolved in DMSO-d6 and analyzed by $^1$H NMR spectroscopy.

**Supplementary Method 5. *In vitro* stability and insulin release studies.**

The stability of Pep/Gal-PNPs *in vitro* was evaluated following the incubation with PBS, simulated gastric fluid (SGF, pH=1.2 with 1% pepsin), and simulated intestinal fluid (SIF, pH=6.8 with 1% trypsin) at 37 °C on a shaker. At predetermined time intervals, the samples were collected, and the particle size, as well as polydispersity index (PDI), was measured.

The insulin release rate of Pep/Gal-PNPs was further measured. The insulin-loaded Pep/Gal-PNPs were incubated in SGF, SIF, and PBS at 1 mg $mL^{-1}$ on a shaker (37 ºC, 50 rpm). At predetermined time intervals, 0.2 mL of sample was removed, and an equal amount of buffer medium was supplemented to maintain the volume. The sample was centrifuged at 9391 × g for 5 min and the released insulin was determined using HPLC. Furthermore, the released insulin was collected and concentrated to 0.5 mg $mL^{-1}$. The conformation of released insulin was analyzed by CD spectroscopy with free insulin solution as a control group.

**Supplementary Method 6. Mucus penetrating studies.**

The E12 cells were seeded on the 12-well transwell plates and continuously cultured for 21 days to produce mucus. The cells were incubated with FITC-labeled Pep/Gal-PNPs at pH 6.8 and 7.4 respectively for 2 h. Then the nanoparticles were removed, and the mucus layer was stained with wheat germ agglutinin (WGA) labeled with Alexa Fluor 555. The distribution of nanoparticles in the mucus was imaged by CLSM and the 3D images were reconstructed by Imaris software (Bitplane AG, Switzerland).

**Supplementary Method 7. Cytotoxicity studies.**

*In vitro* cytotoxicity of nanoparticles was evaluated on the Caco-2 and LO2 cells. In brief, the cells were incubated with Pep/Gal-PNPs at pH 6.8 and 7.4 for 2 h. Then, the cells were washed with PBS and incubated with MTT (0.5 mg $mL^{-1}$, 100 μL) for another 4 h. Followed by discarding the MTT solution, the formazan crystals were

then dissolved in 200 μL DMSO and measured at 590 nm using a microplate reader (n=5).

**Supplementary Method 8. Cellular uptake studies of nanoparticles at different pH.**

The Caco-2 cells were seeded on 24-well plates and cultured for 2 days. The FITC-labeled nanoparticles were diluted with PBS (pH 6.0, 6.5, 7.0, 7.5, and 8.0) to maintain the same dose of encapsulated insulin at 20 ug mL$^{-1}$. Then, the nanoparticles were incubated with Caco-2 cells at 37 °C for 2 h. Then the cells were washed with PBS and disrupted by RIPA lysis buffer. The amount of insulin in the lysate was detected using the microplate reader and the total protein was quantified using the BCA kit.

**Supplementary Method 9. Cellular uptake studies and transcytosis of free insulin.**

The Caco-2 cells were incubated with FITC-insulin at a dose of 20 ug mL$^{-1}$ for 2 h. Then the cells were washed with PBS and disrupted by RIPA lysis buffer. The amount of insulin in the lysate was detected using the microplate reader and the total protein was quantified using the BCA kit. For CLSM observation, the cells were incubated with FITC-insulin for 2 h. Then the cells were washed, fixed with 4% paraformaldehyde, and stained with DAPI for 10 min. The cellular uptake of insulin was observed using CLSM.

The Caco-2 cells were seeded on the 12-well transwell plates and continuously cultured for 21 days. The FITC-insulin was added to Caco-2 cells. Then, 0.2 mL of sample from the basolateral chamber was removed at predetermined time intervals and an equal amount of PBS was supplemented to maintain the volume. The $P_{app}$ value of insulin was calculated according to the equation as described in the main text.

**Supplementary Method 10. Cellular uptake mechanism of Pep/Gal-PNPs.**

To investigate the cellular uptake mechanism of Pep/Gal-PNPs, the Caco-2 cells were preincubated with different endocytic inhibitors including chlorpromazine hydrochloride (30 μM), amiloride (12 μg mL$^{-1}$) and filipin (500 nM) at 37 °C for 1 h. Then the FITC-labeled Pep/Gal-PNPs were added and incubated with cells at pH 6.8 and 7.4 respectively for another 2 h. Additionally, the effect of temperature on cellular uptake was studied by preincubating Caco-2 cells at 4 °C for 1 h. Then the cells were

treated with FITC-labeled Pep/Gal-PNPs at pH 6.8 and 7.4 at 4 °C for another 2 h. The internalization efficiency of nanoparticles was quantified using the BCA kit.

**Supplementary Method 11. ASGPR expression studies.**

The LO2 cells were seeded on 24-well plates and cultured for 2 days. Then the cells were treated with RIPA lysis buffer containing protease inhibitors and the total amount of protein in the lysate was quantified using the BCA kit. The protein lysate was boiled with loading buffer for 10 min and then analyzed using sodium dodecyl sulfate-polyacrylamide gel electrophoresis (SDS-PAGE). After electrophoresis, the gel was stained with Coomassie Blue Staining solution and visualized by a gel imaging system (Gel Doc EZ System, Bio-Rad, USA). For western blot analysis, the protein was further transferred on polyvinylidene fluoride (PVDF) membranes. After blocking with 5% BSA solution for 1 h, the membranes were incubated respectively with anti-ASGPR rabbit pAb (diluted with 5% BSA to 1:50) and anti-GAPDH mouse pAb (diluted with 5% BSA to 1:1000) at 4 °C overnight. Afterward, the membranes were incubated with corresponding horseradish peroxidase (HRP)-conjugated secondary antibodies (diluted with 5% BSA to 1:5000) for 2 h at room temperature. The protein was detected using a western blot detection system (ChemiScope 6000 Exp, Clinx Science Instruments, China) and the protein bands were quantified by ImageJ software.

**Supplementary Method 12. Pulse-chase studies of insulin-loaded Pep/Gal-PNPs on hepatocytes.**

For the pulse-chase assay, the LO2 cells were incubated with free insulin and insulin-loaded Pep-Gal/PNPs with the same dose of insulin at 20 nM for 30 min. Then the cells were washed and incubated with serum-free DMEM medium for predetermined time intervals (10 min, 1 h, and 4 h). The expression of p-AKT in cells was analyzed by western blot as mentioned above.

**Supplementary Method 13. Hepatic insulin availability studies of Pep/Gal-PNPs.**

The diabetic rats were fasted overnight before studies and then administered with free insulin solution at a dose of 5 IU $kg^{-1}$ via subcutaneous injection and insulin-loaded Pep/Gal-PNPs at a dose of 75 IU $kg^{-1}$ via oral gavage. The blood samples were simultaneously collected from the eye vein and portal vein of rats before

administration and at predetermined time intervals after dosing. Then the blood samples were centrifuged at 1503 × g for 10 min, and the collected serum was incubated with acetonitrile for 30 min to release the encapsulated insulin. The serum insulin concentrations were determined using a human insulin ELISA kit.

The hepatic insulin availability ($F_h$) of Pep/Gal-PNPs relative to subcutaneous injection of insulin was measured using the following formula:

$$F_h = \frac{\mathrm{AUC}_{\mathrm{NP,systemic}}}{\mathrm{AUC}_{\mathrm{s.c.,systemic}}} \times \frac{\mathrm{AUC}_{\mathrm{s.c.,portal}}}{\mathrm{AUC}_{\mathrm{NP,portal}}} \quad (2)$$

where $\mathrm{AUC}_{\mathrm{NP,systemic}}$ and $\mathrm{AUC}_{\mathrm{s.c.,systemic}}$ are the systemic (eye vein) AUCs and $\mathrm{AUC}_{\mathrm{NP,portal}}$ and $\mathrm{AUC}_{\mathrm{s.c.,portal}}$ are the portal AUCs following oral administration of Pep/Gal-PNPs and subcutaneous injection of insulin, respectively.

## Supplementary Figures

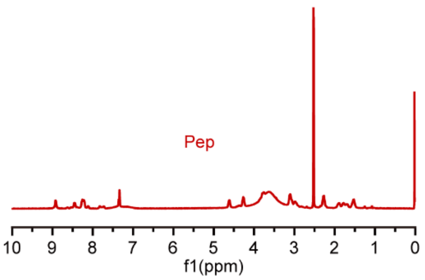


**Supplementary Fig. 1. $^1$H NMR spectrum of Pep.**

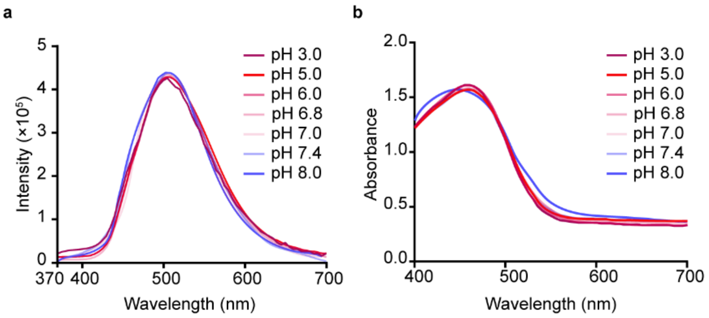


**Supplementary Fig. 2. FRET pair analysis.** a) The emission spectrum of EDANS and b) the absorbance spectrum of Dabcyl under different pH conditions.

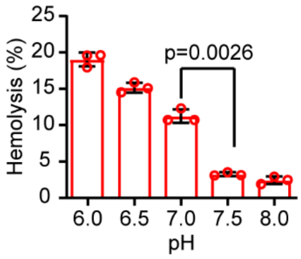


**Supplementary Fig. 3. The hemolytic effect of Pep at different pH.** Data are presented as the mean ± SD (n=3 biologically independent experiments). The statistical analysis was performed using two-tailed Student's t-test, **p = 0.0026.

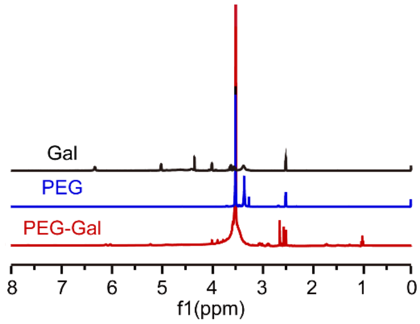


**Supplementary Fig. 4. $^1$H NMR spectra of Gal, PEG, and PEG-Gal polymers.**

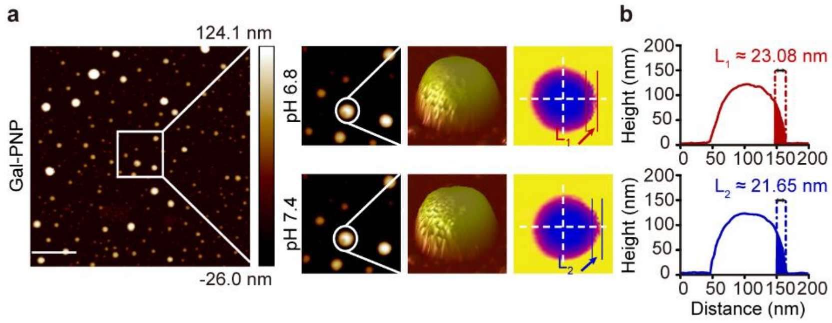


**Supplementary Fig. 5. Detection of Gal-PNPs via AFM.** a) The AFM height map and representative 3D modeling images of Gal-PNPs in pH 6.8 and 7.4 buffer medium. Scale bar: 1 μm. b) Height profiles of Gal-PNPs in pH 6.8 and 7.4 buffer medium. The thickness of ligand corona around nanoparticles was measured via NanoScope Analysis software. Representative images are presented and the data are presented as the mean ± SD (n=3 biologically independent experiments).

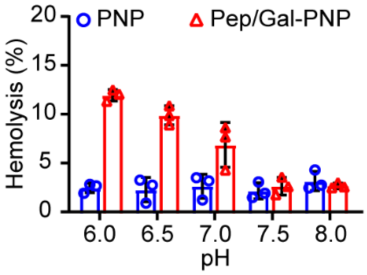


**Supplementary Fig. 6. The hemolytic effects of PNPs and Pep/Gal-PNPs at different pH.** Data are presented as the mean ± SD (n=3 biologically independent experiments).

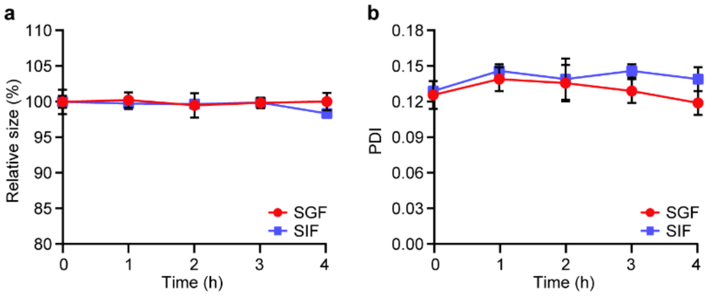


**Supplementary Fig. 7. *In vitro* stability of Pep/Gal-PNPs.** a) The relative size and b) polydispersity intensity (PDI) of Pep/Gal-PNPs in SGF and SIF. Data are presented as the mean ± SD (n=3 biologically independent experiments).

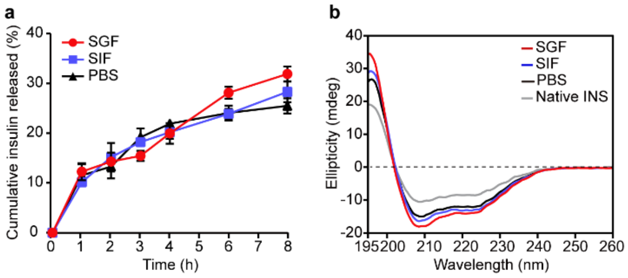


**Supplementary Fig. 8. *In vitro* insulin release from Pep/Gal-PNPs.** a) The *in vitro* insulin release rate of Pep/Gal-PNPs and b) the biological activity of released insulin. Data are presented as the mean ± SD (n=3 biologically independent experiments).

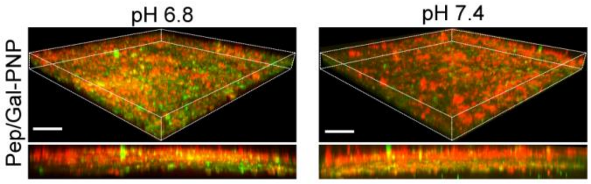


**Supplementary Fig. 9. 3D images showing the distribution of Pep/Gal-PNPs in the mucus layer.** Red: mucus stained with Alexa 555-wheat germ agglutinin; green: FITC-labeled nanoparticles. Scale bar: 20 μm.

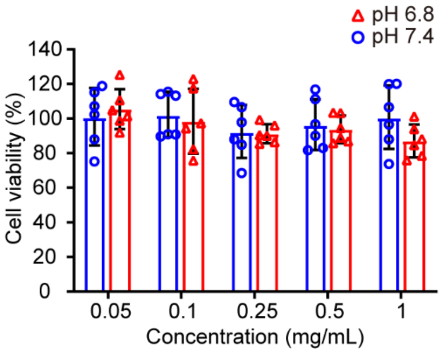


**Supplementary Fig. 10. Cytotoxicity of Pep/Gal-PNPs on Caco-2 cells under different pH conditions.** Data are presented as the mean ± SD (n=6 biologically independent experiments).

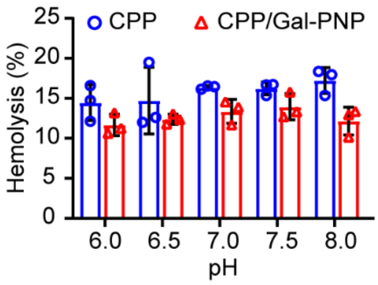


**Supplementary Fig. 11. The hemolytic effects of CPP and CPP/Gal-PNPs at different pH.** Data are presented as the mean ± SD (n=3 biologically independent experiments).

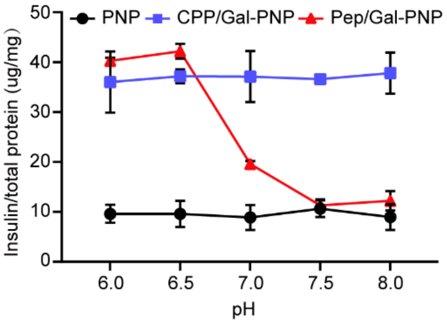


**Supplementary Fig. 12. The cellular uptake of nanoparticles by Caco-2 cells at different pH.** Data are presented as the mean ± SD (n=3 biologically independent experiments).

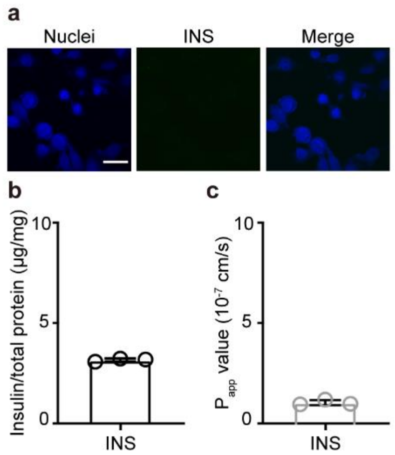


**Supplementary Fig. 13. The cellular uptake and transcytosis of free insulin.** a) CLSM images of cellular uptake of insulin by Caco-2 cells. Blue: nuclei stained with DAPI; green: FITC-insulin. Scale bar: 30 μm. b) Quantitative analysis of insulin internalized by Caco-2 cells. Data are presented as the mean ± SD (n=3 biologically

independent experiments). c) Apparent permeability coefficient ($P_{app}$) value for insulin transport across Caco-2 cells. Data are presented as the mean ± SD (n=3 biologically independent experiments).

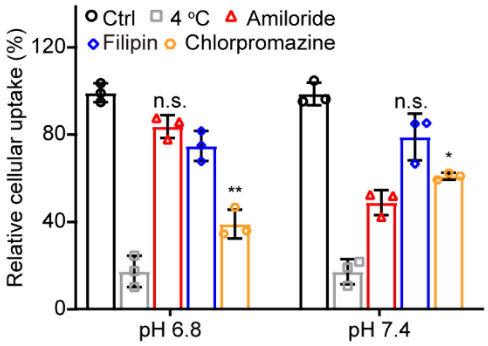


**Supplementary Fig. 14. Cellular uptake mechanism of Pep/Gal-PNPs at different pH.** Relative cellular uptake of Pep/Gal-PNPs by Caco-2 cells in the presence of endocytic inhibitors or under limited conditions (4 °C) compared with control groups. Data are presented as the mean ± SD (n=3 biologically independent experiments). The statistical analysis was performed using two-way analysis of variance (ANOVA) with Dunnett's post-hoc test, n.s., not significant, **p = 0.0031 for chlorpromazine group at pH 6.8, *p = 0.0153 for chlorpromazine group at pH 7.4 compared with the control (Ctrl) group.

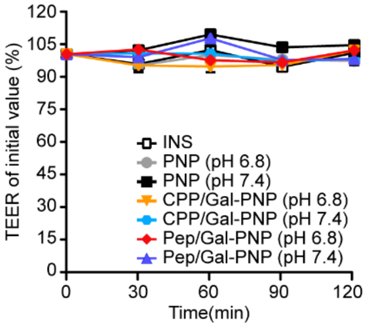


**Supplementary Fig. 15. Transepithelial electrical resistance (TEER) of Caco-2 cells after treatment with different formulations.** Data are presented as the mean ± SD (n=3 biologically independent experiments).

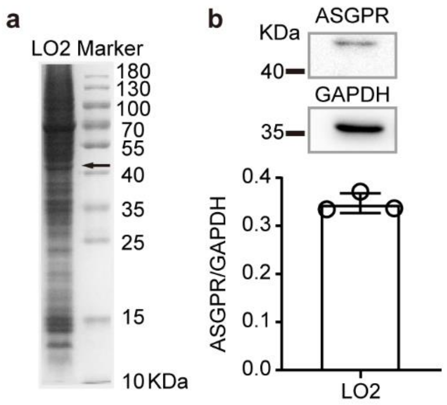


**Supplementary Fig. 16. The expression of ASGPRs on liver cells.** a) SDS-PAGE analysis of ASGPR expression on LO2 cells. The black arrow denotes the expression of ASGPR. b) Western blot analysis and quantitative analysis of ASGPR expression on LO2 cells. The samples derived from the same experiment and blots were processed in parallel. Data are presented as the mean ± SD (n=3 biologically independent experiments).

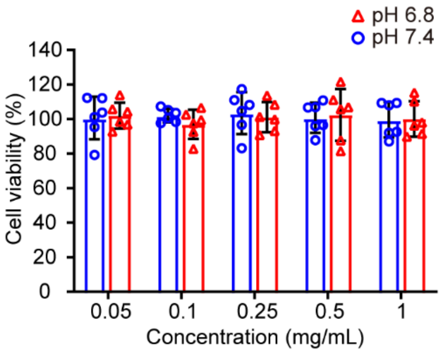


**Supplementary Fig. 17. Cytotoxicity of Pep/Gal-PNPs on LO2 cells under different pH conditions.** Data are presented as the mean ± SD (n=6 biologically independent experiments).

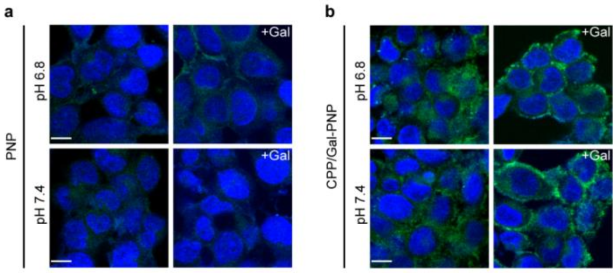


**Supplementary Fig. 18. CLSM images of a) PNPs and b) CPP/Gal-PNPs binding to LO2 cells.** +Gal: in the presence of free galactose. Scale bar: 10 µm.

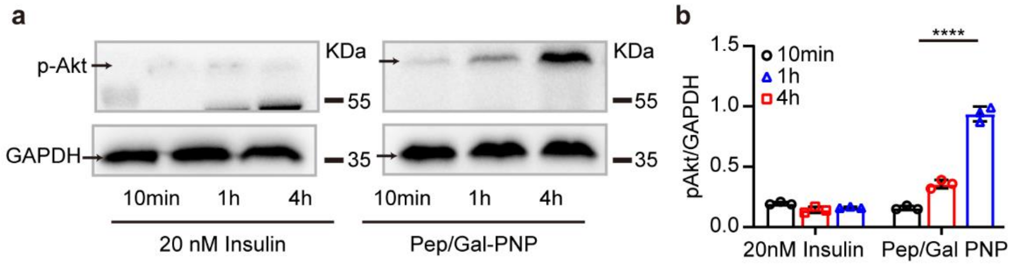


**Supplementary Fig. 19. Pulse-chase analysis of p-AKT expression in LO2 cells. a)** Western blot analysis and b) quantitative analysis of the p-AKT expression in LO2 cells. The samples derived from the same experiment and blots were processed in parallel. Data are presented as the mean ± SD (n=3 biologically independent experiments). The statistical analysis was performed using two-tailed Student's t-test, ****$p < 0.0001$.

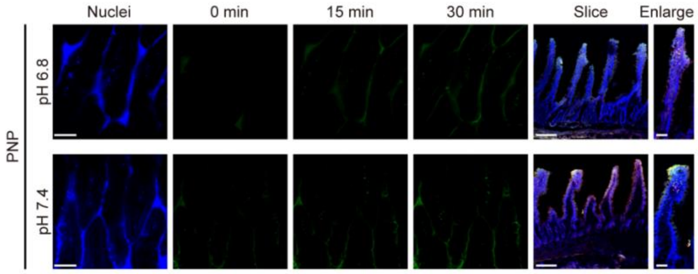


**Supplementary Fig. 20. Intestinal absorption of PNPs at different pH.** The TPM images presented the absorption of PNPs in the intestinal villi of rats. Scale bar: 100 µm. And the CLSM images of intestinal villi sections from rats after administration with PNPs for 2 h. Blue: villi stained with DAPI; green: FITC-labeled nanoparticles; yellow: the colocalization signals of FITC and RITC loaded in PNPs. Scale bars: intestinal slice images, 200 µm; enlarged images, 50 µm.

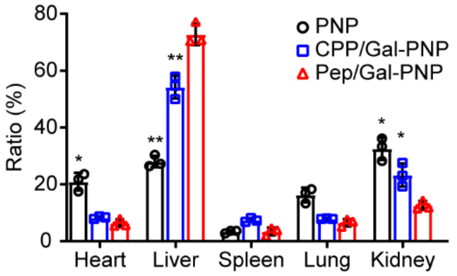


**Supplementary Fig. 21. Quantitative analysis of accumulation ratio of PNPs, CPP/Gal-PNPs, and Pep/Gal-PNPs in major organs.** Data are presented as the

mean ± SD (n=3 biologically independent experiments). The statistical analysis was performed using two-way analysis of variance (ANOVA) with Dunnett's post-hoc test. PNP group: *p = 0.243 for heart, **p = 0.0073 for liver, *p = 0.0191 for kidney; CPP/Gal-PNP group: **p= 0.0083 for liver, *p = 0.0362 for kidney compared with the Pep/Gal-PNP group.

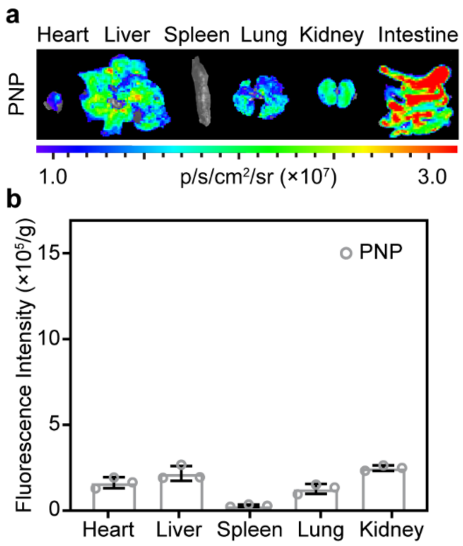


**Supplementary Fig. 22. *In vivo* distribution of PNPs.** a) The accumulation of PNPs in major rat organs as imaged by IVIS. b) The fluorescence intensity of different tissue homogenates prepared from rats 4 h after the oral administration of FITC-labeled PNPs. Data are presented as the mean ± SD (n=3 biologically independent experiments).

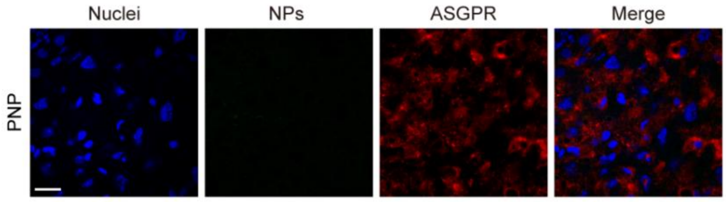


**Supplementary Fig. 23. CLSM images of colocalization of PNPs with ASGPRs in liver sections.** Blue: nuclei stained with DAPI; green: FITC-labeled PNPs; red: antibody-labeled ASGPRs; yellow: the colocalization signals. Scale bar: 20 μm.

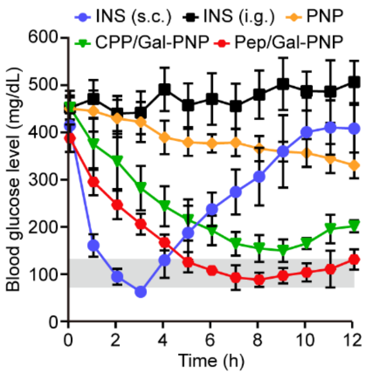


**Supplementary Fig. 24. Blood glucose level versus time profiles in type I diabetic rats.** The rats were treated with oral administration of insulin (INS, i.g., 75 IU $kg^{-1}$), insulin-loaded nanoparticle formulations (PNP, CPP/Gal-PNP and Pep/Gal-PNP, i.g., 75 IU $kg^{-1}$), and subcutaneous injection of insulin (INS, s.c., 5 IU $kg^{-1}$). The gray box denotes the normal blood glucose range (70-140 mg $dL^{-1}$). Data are presented as the mean ± SD (n=6 biologically independent rats).

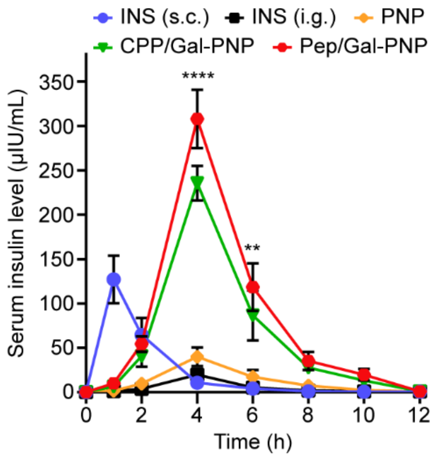


**Supplementary Fig. 25. Portal serum insulin level versus time profiles in type I diabetic rats treated with different formulations.** Data are presented as the mean ± SD (n=6 biologically independent rats). The statistical analysis was performed using two-way analysis of variance (ANOVA) with Turkey's post-hoc test. ****$p < 0.0001$ at 4 h, **$p = 0.0014$ at 6 h compared with the CPP/Gal-PNP group.

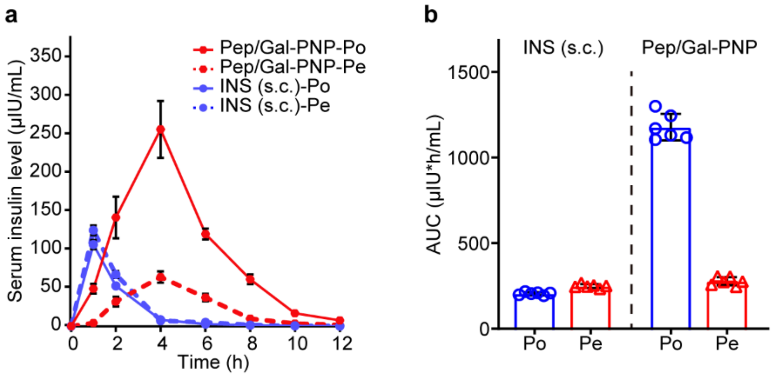


**Supplementary Fig. 26. Simultaneous measurement of portal (Po) and peripheral (Pe) a) serum insulin level versus time profiles and b) the total area under the serum insulin level versus time curve in type I diabetic rats following oral administration of insulin-loaded Pep/Gal-PNP (75 IU kg$^{-1}$) and subcutaneous injection of insulin (5 IU kg$^{-1}$).** Data are presented as the mean ± SD (n=6 biologically independent rats).

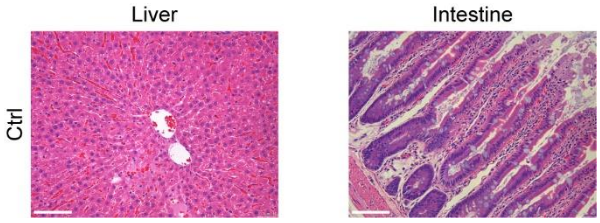


**Supplementary Fig. 27. The H&E staining images of liver and intestine sections from healthy rats treated with PBS.** Scale bar: 100 μm.

**Supplementary Tables**

**Supplementary Table 1. Characterization of nanoparticles.** Data are presented as the mean ± SD (n=3).

| Group | Size (nm) | Zeta Potential (mV) | EE[a] (%) | LC[b] (%) | Pep/CPP modification rate (%) | Gal modification rate (%) |
|---|---|---|---|---|---|---|
| PNP | 108.6 ± 1.9 (pH 6.8)<br>107.2 ± 3.6 (pH 7.4) | -33.8 ± 2.3 (pH 6.8)<br>-37.4 ± 2.9 (pH 7.4) | 46.3 | 7.5 | - | - |
| Gal-PNP | 129.1 ± 4.3 (pH 6.8)<br>128.0 ± 2.7 (pH 7.4) | -33.6 ± 2.6 (pH 6.8)<br>-35.4 ± 2.5 (pH 7.4) | 53.8 | 8.4 | - | 5.2 |
| Pep-PNP | 135.0 ± 3.8 (pH 6.8)<br>117.6 ± 4.0 (pH 7.4) | 24.7 ± 2.1 (pH 6.8)<br>-30.8 ± 1.4 (pH 7.4) | 42.1 | 6.8 | 4.7 | - |
| CPP/Gal-PNP | 126.8 ± 2.4 (pH 6.8)<br>126.2 ± 3.9 (pH 7.4) | 28.3 ± 1.7 (pH 6.8)<br>27.4 ± 2.4 (pH 7.4) | 44.5 | 7.2 | 4.9 | 5.8 |
| Pep/Gal-PNP | 136.1 ± 3.1 (pH 6.8)<br>126.5 ± 1.1 (pH 7.4) | 25.1 ± 3.3 (pH 6.8)<br>-28.2 ± 3.9 (pH 7.4) | 48.1 | 7.9 | 5.4 | 5.6 |

[a] EE: Entrapment efficiency; [b] LC: Loading capacity.

**Supplementary Table 2. Pharmacodynamic parameters of different insulin formulations following oral or subcutaneous administration to diabetic rats.** Data are presented as the mean ± SD (n=6).

| | Insulin (s.c.) | Insulin (i.g.) | PNP (i.g.) | CPP/Gal-PNP (i.g.) | Pep/Gal-PNP (i.g.) |
|---|---|---|---|---|---|
| AAC (μIU*h $mL^{-1}$)[a] | 464.4 | - | 169.0 | 577.6 | 705.0 |
| PA (%)[b] | 100 | - | 2.4 | 8.3 | 10.1 |

[a] AAC: area above the blood glucose level versus time curve; [b] PA: relative pharmacological bioavailability.

**Supplementary Table 3. Measurement of portal serum insulin levels in diabetic rats following oral or subcutaneous administration of different insulin formulations.** Data are presented as the mean ± SD (n=6).

| | Insulin (s.c.) | Insulin (i.g.) | PNP (i.g.) | CPP/Gal-PNP (i.g.) | Pep/Gal-PNP (i.g.) |
|---|---|---|---|---|---|
| Dose (IU $kg^{-1}$) | 5 | 75 | 75 | 75 | 75 |
| AUC (μIU*h $mL^{-1}$)[a] | 257.0 ± 28.8 | 62.9 ± 11.5 | 150.0 ± 21.1 | 794.5 ± 51.9 | 1056.0 ± 63.3 |
| Ratio[b] | 4.1 | - | 2.4 | 12.6 | 16.8 |

[a] AUC: area under the portal serum insulin level versus time curve; [b] Ratio: the increased AUC ratio compared to Insulin (i.g.) group.
