# Supplementary material for: Ligand-switchable nanoparticles resembling viral surface for sequential drug delivery and improved oral insulin therapy
Source: Nat Commun. 2022 Nov 4;13:6649. doi: 10.1038/s41467-022-34357-8 (PMC9636268; doi:10.1038/s41467-022-34357-8)
Supplement: Supplementary file 3 — Reporting Summary [file 41467_2022_34357_MOESM3_ESM.pdf]

nature portfolio

Corresponding author(s): Gang Wei and Yong Gan

Last updated by author(s): Oct 9, 2022

# Reporting Summary

Nature Portfolio wishes to improve the reproducibility of the work that we publish. This form provides structure for consistency and transparency in reporting. For further information on Nature Portfolio policies, see our Editorial Policies and the Editorial Policy Checklist.

## Statistics

For all statistical analyses, confirm that the following items are present in the figure legend, table legend, main text, or Methods section.

| n/a | Confirmed | |
|---|---|---|
| [ ] | [x] | The exact sample size ($n$) for each experimental group/condition, given as a discrete number and unit of measurement |
| [ ] | [x] | A statement on whether measurements were taken from distinct samples or whether the same sample was measured repeatedly |
| [ ] | [x] | The statistical test(s) used AND whether they are one- or two-sided<br>*Only common tests should be described solely by name; describe more complex techniques in the Methods section.* |
| [x] | [ ] | A description of all covariates tested |
| [ ] | [x] | A description of any assumptions or corrections, such as tests of normality and adjustment for multiple comparisons |
| [ ] | [x] | A full description of the statistical parameters including central tendency (e.g. means) or other basic estimates (e.g. regression coefficient) AND variation (e.g. standard deviation) or associated estimates of uncertainty (e.g. confidence intervals) |
| [ ] | [x] | For null hypothesis testing, the test statistic (e.g. $F$, $t$, $r$) with confidence intervals, effect sizes, degrees of freedom and $P$ value noted<br>*Give P values as exact values whenever suitable.* |
| [x] | [ ] | For Bayesian analysis, information on the choice of priors and Markov chain Monte Carlo settings |
| [x] | [ ] | For hierarchical and complex designs, identification of the appropriate level for tests and full reporting of outcomes |
| [x] | [ ] | Estimates of effect sizes (e.g. Cohen's $d$, Pearson's $r$), indicating how they were calculated |

*Our web collection on statistics for biologists contains articles on many of the points above.*

## Software and code

Policy information about availability of computer code

| | |
|---|---|
| Data collection | Data were collected using the software described in each experiment.<br>The size and zeta potential data of nanoparticles were collected using Zetasizer Nano software (v 3.30); The atomic force microscopy (AFM) data were collected using NanoScope Analysis software (v 940r1); The fluorescence intensity and fluorescence resonance energy transfer (FRET) data were collected using BioTek Gen5 software (v 2.0); The bright field microscopy images were captured using Leica Application Suite X software (v 3.3.0); The confocal laser scanning microscopy (CLSM) images were captured using Olympus FV10-ASW Viewer software (v 4.2) and Leica Application Suite software (v 3.5); The two-photon microscopy (TPM) images were captured using Olympus FV10-ASW Viewer software (v 4.2); The accumulation of nanoparticles in major rat organs was imaged using IVIS spectrum system (Perkin Elmer, USA) with Living Image software (v 4.0); The confocal laser endomicroscopy (CLE) images were captured using Optiscan Imager software (v B30). |
| Data analysis | Graphpad Prism (v 7.03) was used for statistical analysis; Image J (v 1.8.0) was used for images analysis; CChemDraw (Version 14.0) was used for chemical formula; MestReNova (v 12.0.0) was used for proton nuclear magnetic resonance (1H NMR) data analysis; Spectra Manager software (v 2.08.04) was used for circular dichroism (CD) data analysis; NanoScope Analysis software (v 1.8) was used for atomic force microscopy (AFM) data analysis; Imaris software (v 7.2.3) was used for analyzing the colocalization coefficient (R). |

For manuscripts utilizing custom algorithms or software that are central to the research but not yet described in published literature, software must be made available to editors and reviewers. We strongly encourage code deposition in a community repository (e.g. GitHub). See the Nature Portfolio guidelines for submitting code & software for further information.

## Data

Policy information about availability of data

All manuscripts must include a data availability statement. This statement should provide the following information, where applicable:

- Accession codes, unique identifiers, or web links for publicly available datasets
- A description of any restrictions on data availability
- For clinical datasets or third party data, please ensure that the statement adheres to our policy

The source data underlying Figs. 3-6, Supplementary Figs. 3, 5-8, 10-17, 19, 21-22, 24-26 and Supplementary Table 1 are provided in “Source Data” file. All the other data that support the findings of this study are available within the Article and its Supplementary Information files and from the corresponding author upon reasonable request. Source data are provided with this paper.

## Human research participants

Policy information about studies involving human research participants and Sex and Gender in Research.

| | |
|---|---|
| Reporting on sex and gender | Not available. |
| Population characteristics | Not available. |
| Recruitment | Not available. |
| Ethics oversight | Not available. |

Note that full information on the approval of the study protocol must also be provided in the manuscript.

# Field-specific reporting

Please select the one below that is the best fit for your research. If you are not sure, read the appropriate sections before making your selection.

☒ Life sciences ☐ Behavioural & social sciences ☐ Ecological, evolutionary & environmental sciences

For a reference copy of the document with all sections, see nature.com/documents/nr-reporting-summary-flat.pdf

# Life sciences study design

All studies must disclose on these points even when the disclosure is negative.

| | |
|---|---|
| Sample size | Sample size was chosen to ensure reproducibility of the experiments and to support meaningful conclusions. The sample size (n) of each experiment is provided in Figure legends in the Article and its Supplementary Information. |
| Data exclusions | On principle, data were only excluded for failed experiments, such as negative results on our positive control. |
| Replication | All of the experimental findings could be reliably reproduced. All experiments were replicated multiple times with reproducible results as indicated in the figure legends. In animal experiments, at least six rats were included in each group. |
| Randomization | The simple randomization method was applied to allocate samples and animals into different groups. |
| Blinding | No blinding experiments were designed in this experiment. Blinding was not feasible for in vivo administration. All data collected was quantifiable and blinding would not change any bias in data collected. |

# Reporting for specific materials, systems and methods

We require information from authors about some types of materials, experimental systems and methods used in many studies. Here, indicate whether each material, system or method listed is relevant to your study. If you are not sure if a list item applies to your research, read the appropriate section before selecting a response.

## Materials & experimental systems

| n/a | Involved in the study | |
|---|---|---|
| ☐ | ☒ | Antibodies |
| ☐ | ☒ | Eukaryotic cell lines |
| ☒ | ☐ | Palaeontology and archaeology |
| ☐ | ☒ | Animals and other organisms |
| ☒ | ☐ | Clinical data |
| ☒ | ☐ | Dual use research of concern |

## Methods

| n/a | Involved in the study | |
|---|---|---|
| ☒ | ☐ | ChIP-seq |
| ☒ | ☐ | Flow cytometry |
| ☒ | ☐ | MRI-based neuroimaging |

# Antibodies

Antibodies used

All the antibodies were diluted and used following the supplier's protocols.
Anti-ASGPR rabbit antibody (Sangon Biotechnology, D121918), used at 1:50 from 0.3 mg/mL stock solution.
Anti-GAPDH mouse antibody (Sangon Biotechnology, D190090), used at 1:1000 from 1 mg/mL stock solution.
Phospho-AKT (Ser473) rabbit antibody (Bimake, A5030), used at 1:1000 from 1 mg/mL stock solution.
HRP-conjugated goat anti-rabbit IgG (Sangon Biotechnology, D110058), used at 1:5000 from 0.2 mg/mL stock solution.
HRP-conjugated goat anti-mouse IgG (Sangon Biotechnology, D110087), used at 1:5000 from 0.2 mg/mL stock solution.
Alexa 647 labeled goat anti-rabbit IgG (Yeasen Biotechnology, 33113ES60), used at 1:200 from 0.75 mg/mL stock solution.

Validation

Anti-ASGPR rabbit antibody (Sangon Biotechnology, D121918) has been validated for use in immunohistochemistry analysis on human liver cancer and human colon cancer and western blot studies, as stated on the Sangon Biotechnology product page.
Anti-GAPDH mouse antibody (Sangon Biotechnology, D190090) has been validated for use in immunohistochemistry analysis on human tonsil tissue and human thyroid cancer and western blot studies, as stated on the Sangon Biotechnology product page.
Phospho-AKT (Ser473) rabbit antibody (Bimake, A5030) has been validated for use in immunohistochemistry analysis on mouse brain tissue and western blot studies, as stated on the Bimake product page. This antibody was also validated in previous publication (Zhang NN, et al. Cell Prolif. 2020, 53(6): e12825.).

# Eukaryotic cell lines

Policy information about cell lines and Sex and Gender in Research

Cell line source(s)

Cell line sources were provided under "Materials and Methods: Cell culture" section.
Human colorectal adenocarcinoma cells (Caco-2) and human fetal hepatocytes (LO2) were purchased from the American Type Culture Collection (ATCC, Manassas, USA). HT29-MTX-E12 (E12) cell line was kindly provided by Novo Nordisk A/S (Denmark).

Authentication

The cell lines were certificated by the manufactures.
Mucus-secreting ability of E12 cells was authenticated by using Alexa Fluor 555 conjugated wheat germ agglutinin (WGA) to stain mucus (see Supplementary Figure 9).
The permeability of Caco-2 cells was authenticated by measuring transepithelial electrical resistance (TEER) values (see Supplementary Figure 15).
Expression of asialoglycoprotein receptors (ASGPRs) on LO2 cells was authenticated using western blot studies (see Supplementary Figure 16).

Mycoplasma contamination

All cell lines were tested negative for mycoplasma contamination.

Commonly misidentified lines
(See ICLAC register)

No commonly misidentified cell lines were used.

# Animals and other research organisms

Policy information about studies involving animals; ARRIVE guidelines recommended for reporting animal research, and Sex and Gender in Research

Laboratory animals

As reported in the "Materials and Methods: Animal care" section, 6~8 weeks male Sprague–Dawley (SD) rats (200–220 g) were provided by the Animal Experiment Center of Shanghai Institute of Materia Medica (Shanghai, China), Chinese Academy of Sciences.

Wild animals

The study did not involve wild animals.

Reporting on sex

The study used only male rats.

Field-collected samples

The study did not involve samples collected from the field.

Ethics oversight

All animal experiments were conducted following the relevant requirements of the Institutional Animal Care and Use Committee (IACUC) guidelines of the Shanghai Institute of Materia Medica, Chinese Academy of Sciences.

Note that full information on the approval of the study protocol must also be provided in the manuscript.
